# Retraction: Tree bark scrape fungus: A potential source of laccase for application in bioremediation of non-textile dyes

**DOI:** 10.1371/journal.pone.0328188

**Published:** 2025-07-16

**Authors:** The PLOS One Editors

The *PLOS One* Editors retract this article [1, corrected in 2] because it was identified as one of a series of submissions for which we have concerns about potential manipulation of the publication process, authorship, competing interests, and peer review. We regret that the issues were not addressed prior to the article’s publication.

RZS and HAEE did not agree with the retraction. HMB, S, NM, AME, AS, and DJD either did not respond directly or could not be reached.
